# Genome-Wide Identification and Expression Analysis of Aquaporin Gene Family in 84K Poplar

**DOI:** 10.3390/plants15203167

**Published:** 2026-10-16

**Authors:** Mingqiang Zhu, Kaiqi Wang, Jiyu Wang, Kuan Sun, Haiyang Wang, Yunfeng Li, Jin Wang, Shunqin Zhu

**Affiliations:** 1 Integrative Science Center of Germplasm Creation in Western China (CHONGQING) Science City, Chongqing Technology Innovation Center of Breeding, Chongqing Key Laboratory of Plant Resource Conservation and Germplasm Innovation, School of Life Sciences, Southwest University, Chongqing 400715, China; zmqjukey@whu.edu.cn (M.Z.);; 2 Chongqing Nanshan Botanical Garden, Chongqing 400715, China; 3 Key Laboratory of Eco-Environments of Three Gorges Reservoir Region, Ministry of Education, School of Life Sciences, Southwest University, Chongqing 400715, China

**Keywords:** 84K poplar, aquaporin, gene family, expression pattern, drought stress response

## Abstract

Aquaporins (AQPs) mediate the transmembrane transport of water and diverse small molecules, playing essential roles in plant growth and development and stress responses. However, their genome-wide organization and evolution at the subgenome level in hybrid 84K poplar (*Populus alba* × *Populus tremula* var. *glandulosa*) remain incompletely characterized. Based on the telomere-to-telomere genome of 84K poplar, we identified 94 *PagAQPs*, with 46 and 48 members in the A and G subgenomes, respectively. Phylogenetic analysis classified them into five subfamilies unevenly distributed across 29 chromosomes, with PagPIP and PagTIP being the two largest subfamilies. Most *PagAQPs* were retained as corresponding A/G allelic pairs, whereas few exhibit subgenome-specific retention. Valid Ka/Ks ratios for 38 allelic pairs and 59 paralogous gene pairs were all below 1, indicating predominant purifying selection. Gene structure, conserved motifs, and substrate-recognition residues showed conservation within subfamilies and divergence among subfamilies. The promoters contained predicted cis-acting elements associated with light, phytohormone and abiotic stress responses. Expression profiling showed that some *PagPIP* and *PagTIP* members were highly expressed in vascular tissues and exhibited changes in transcript abundance under nitrogen and drought treatments. Compared with wild-type plants, the *35S*:*PagPIP2;6A* overexpression lines were shorter before treatment and exhibited higher leaf relative water content and lower malondialdehyde content after water withholding. This study systematically characterized the subgenomic distribution, evolutionary features and expression patterns of the PagAQP family in 84K poplar and identified candidate genes for further functional investigation.

## 1. Introduction

Water is the primary limiting factor for plant growth and development, and its precise transmembrane transport regulation is crucial for maintaining cell turgor pressure, photosynthetic efficiency and stress adaptation capacity [1,2]. Plant AQPs are core members of the major intrinsic proteins (MIPs) superfamily, which are channel proteins that efficiently and selectively mediate the transmembrane transport of water molecules, as well as small neutral solutes including glycerol, urea, ammonia, carbon dioxide and hydrogen peroxide [3,4,5,6]. Extensive studies have demonstrated that AQPs not only participate in fundamental physiological processes including root water uptake, long-distance vascular water transport, stomatal movement, photosynthesis, and cell elongation, but also play critical regulatory roles in plant responses to abiotic stresses such as drought, low temperature, and nutrient deficiency [7,8,9,10].

At present, the AQP gene family has been systematically identified in numerous plant species. For example, 35 AQP members have been identified in *Arabidopsis thaliana* [11], 33 in *Oryza sativa* [12], 86 in *Glycine max* [13], 61 in *Camellia sinensis* [14], and 55 in *Populus trichocarpa* [15]. These studies revealed that plant AQP gene families are highly conserved throughout evolution and can generally be classified into four or five subfamilies: plasma membrane intrinsic protein (PIP), tonoplast intrinsic protein (TIP), NOD26-like intrinsic protein (NIP), small basic intrinsic protein (SIP), and X intrinsic proteins (XIP), which have been found only in certain dicotyledonous plants but have been lost during the evolution of monocots and *Brassicaceae* [11,12,13,14,15,16]. Distinct AQP subfamilies exhibit unique subcellular localization, substrate selectivity and expression regulation patterns, which together form an intricate network for water and solute transport in plants [17]. The structure of AQPs is highly conserved, usually composed of six transmembrane helices connected by five loop regions (LA-LE), and its N-terminus and C-terminus are located inside the cytoplasm [18]. Two conserved asparagine–proline–alanine (NPA) motifs are separately distributed in Loop B and Loop E, which determine the selective water permeability of AQPs [19,20]. Residues of the H2, H5, LE1 and LE2 loops collectively constitute the aromatic/arginine (Ar/R) selectivity filter, which fine-tunes substrate permeability and transport specificity [21]. Moreover, five conserved Froger sites, P1 in Loop C, P2 and P3 in Loop E, P4 and P5 in TM6, are crucial for regulating channel specificity [22].

Among woody plants, *Populus* species serve as model trees for forest functional genomics research, and their AQP gene families have been extensively investigated. A total of 55 aquaporin genes have been identified in the *Populus trichocarpa* genome, comprising 15 PIPs, 17 TIPs, 11 NIPs, 6 SIPs and 6 XIPs, representing remarkable family expansion relative to species such as *Arabidopsis thaliana* and *Oryza sativa* [15]. Further investigations have uncovered prominent expansion and extensive polymorphism of XIP members in *Populus*. Nevertheless, only *PtXIP2;1* and *PtXIP3;2* have been found to be expressed in vegetative tissues and to respond to drought, salicylic acid and mechanical wounding [23]. In addition, some AQP members in *Populus trichocarpa* exhibit tissue- and environment-specific expression, and the duplicated genes undergo obvious functional divergence [24]. Physiological and functional analyses of poplar *PtPIPs* demonstrated that *PtPIPs* participate in regulating root hydraulic conductivity, responding to transpiration demand, and repairing xylem embolism [25,26]; among them, *PtoPIP1;1* is involved in the response to osmotic stress and facilitates cell growth by modulating plant water status and cell turgor [27,28]. Furthermore, 22 AQP family members have been identified in the *Populus euphratica* genome, whereas no XIP members were detected, and these members display differential expression responses to drought, salinity and phytohormone treatments [29]. Collectively, these studies have revealed, at the haplotype-genome level, differences in the composition of AQP gene family members among different *Populus* species and have also demonstrated the important roles of AQPs in stress adaptation. However, the evolutionary patterns and functional divergence characteristics of AQPs derived from different parental genomes in diploid hybrid poplar remain entirely unknown.

84K poplar is an elite hybrid poplar widely cultivated in North and Northwest China, exhibiting heterotic characteristics including fast growth, superior wood properties, and strong stress tolerance, conferring great application value in shelter forest construction and short-rotation industrial timber plantation cultivation, and is also widely utilized in poplar molecular biology and genetic improvement research [30,31,32]. The 84K poplar genome contains two subgenomes (subgenome A and subgenome G) derived from its two parents, and the recent release of its haplotype-phased telomere-to-telomere (T2T) genome has provided a valuable genomic resource for deciphering the evolutionary patterns and functional divergence of gene families in hybrid poplars at the subgenomic level [33]. However, the AQP gene family in 84K poplar remains poorly understood with regard to its genomic composition, A/G-subgenome distribution, retention of parental gene copies, evolutionary conservation, promoter variation, and expression patterns across tissues and abiotic stress conditions.

In this study, based on the T2T genome and available transcriptome data of 84K poplar, we systematically identified members of the AQP gene family derived from the A and G subgenomes. We analyzed their phylogenetic relationships, physicochemical properties, chromosomal and subgenomic distributions, collinearity, gene structures, conserved motifs, substrate-recognition residues, and promoter cis-acting elements. We further examined tissue-expression profiles and transcriptional responses of *PagAQPs* to drought, nitrogen, and salt treatments using transcriptomic data and qRT-PCR, and conducted a preliminary functional assessment of *PagPIP2;6A* by generating overexpression lines. These findings provide a subgenomic level view of the composition, parental gene retention patterns, evolutionary conservation, and expression patterns of the PagAQP family and identify candidate genes for further investigation of water and nutrient transport and stress responses in hybrid poplar.

## 2. Results

### 2.1. Genome-Wide Identification and Phylogenetic Analysis of AQP Gene Family in 84K Poplar

**Figure 1:**
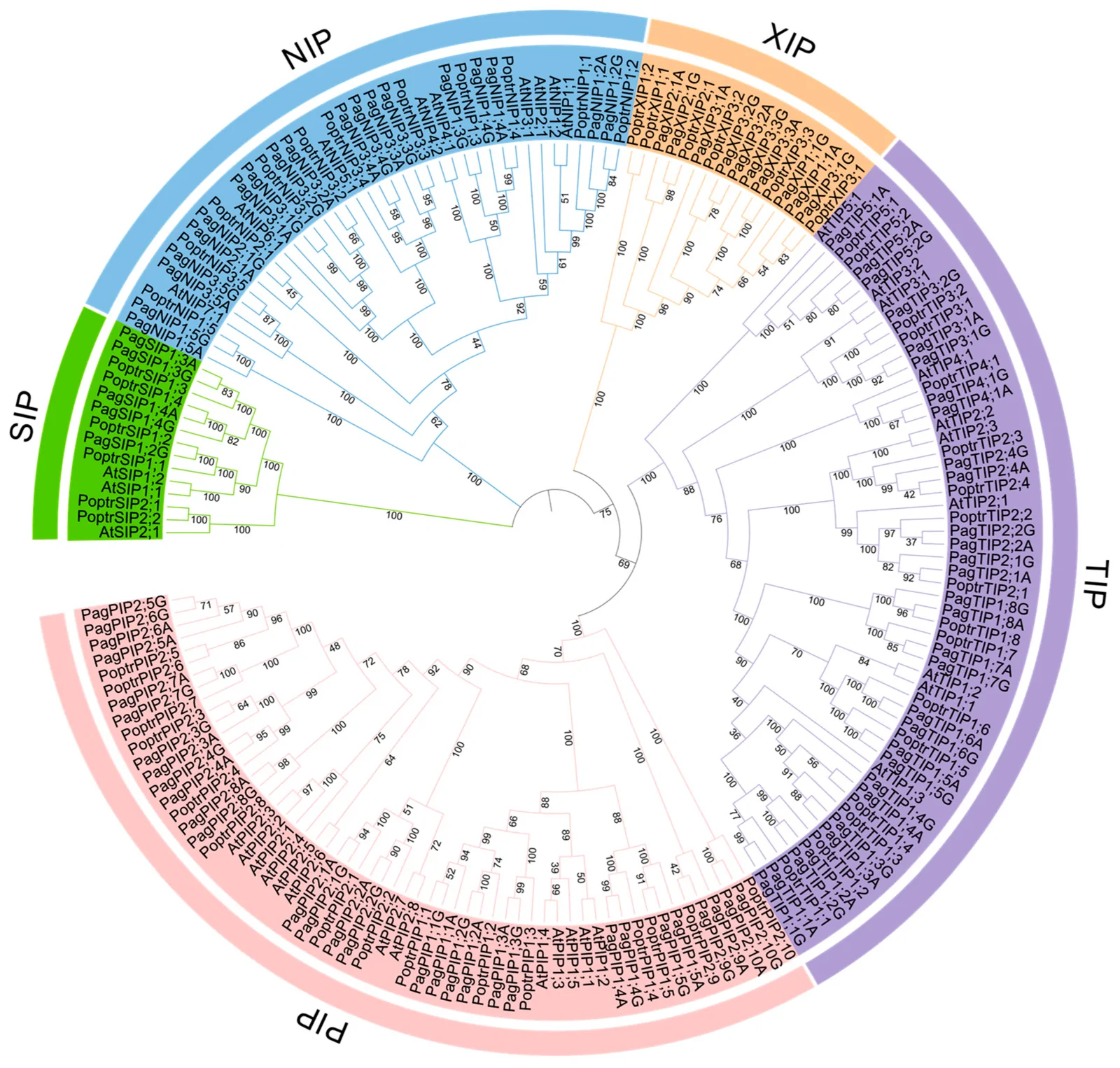
Phylogenetic analysis of AQP proteins in 84K poplar, *Arabidopsis thaliana* and *Populus trichocarpa*. There were 94 PagAQPs in 84K poplar, 35 AtAQPs in *Arabidopsis thaliana*, and 55 PoptrAQPs in *Populus trichocarpa*. Different background colors represent five subfamilies: red represents PIP subfamily, purple represents TIP subfamily, blue represents NIP subfamily, green represents SIP subfamily, yellow represents XIP subfamily.

To identify the members of the AQP gene family in the 84K poplar genome, we used the protein sequences of AQPs from *Arabidopsis thaliana* and *Populus trichocarpa* as queries to perform BLASTp searches against the protein database of 84K poplar, and an HMM search was conducted using the HMM profile (PF00230). A total of 94 PagAQPs were identified, including 46 and 48 members in the A and G subgenomes, respectively (Table S1), indicating no substantial difference in the overall number of PagAQPs between the two subgenomes. Phylogenetic analysis of AQPs from 84K poplar, *Arabidopsis thaliana* and *Populus trichocarpa* showed that all AQPs were divided into five subfamilies (TIP, PIP, NIP, SIP, and XIP) (Figure 1), which is consistent with previously reported AQPs classification in other angiosperms [14,15]. In the 84K poplar genome, the PagPIP and PagTIP subfamilies contained the largest numbers of members, each accounting for 31.91% of all PagAQPs. The numbers of PagPIP and PagTIP subfamilies in the A/G subgenomes were 15/15 and 15/15, respectively, whereas those of PagNIP, PagXIP, and PagSIP subfamilies were 9/10, 5/5, and 2/3, respectively (Figure 1; Table S1). Compared with the five AQP subfamilies in the haplotype genome of *Populus trichocarpa*, namely PoptrPIPs (15), PoptrTIPs (17), PoptrNIPs (11), PoptrXIPs (6), and PoptrSIPs (6) [15], all four subfamilies except the PagPIP subfamily contained fewer members in each subgenome of 84K poplar. Collectively, the PagAQP gene family showed broadly balanced membership between the A and G subgenomes, and its subfamily composition was consistent with that of *Populus trichocarpa*. However, except for PagPIP, each of the other four subfamilies contained fewer members in both subgenomes than in the *Populus trichocarpa* haplotype genome, indicating differential gene retention among AQP subfamilies during *Populus* evolution.

The analysis of the characteristic parameters of all PagAQPs in 84K poplar showed that the protein length of PagAQPs ranged from 210 residues (PagSIP1;2G) to 562 residues (PagXIP3;1A), and the corresponding molecular weights (MW) ranged from 22,007 Da to 61,199.11 Da. The predicted isoelectric points (pI) ranged from 4.67 to 9.67. Interestingly, the pI values of most members of the PagPIP, PagXIP, PagNIP, and PagSIP subfamilies were more than 7, whereas the pI values of most members of the PagTIPs subfamily were less than 7, indicating that these PagTIP subfamily members are acidic. Except for PagPIP2;10G, PagNIP1;5A, PagNIP1;5G, PagNIP3;5A and PagNIP3;5G, the instability indices (II) of the remaining PagAQPs were less than 40, indicating that PagAQPs had higher structural stability. The aliphatic index (AI) ranged from 77.78 to 129.29. The grand average of hydropathicity (GRAVY) values of PagAQPs ranged from 0.024 to 1.06, indicating that these proteins were predominantly hydrophobic, although their hydropathicity varied considerably among individual members. Subcellular localization prediction showed that PagAQPs were predominantly localized to the plasma membrane and vacuole. In addition, most PagAQPs contained more than five transmembrane domains (Table S1).

### 2.2. Distribution of PagAQPs on Chromosomes in 84K Poplar

**Figure 2:**
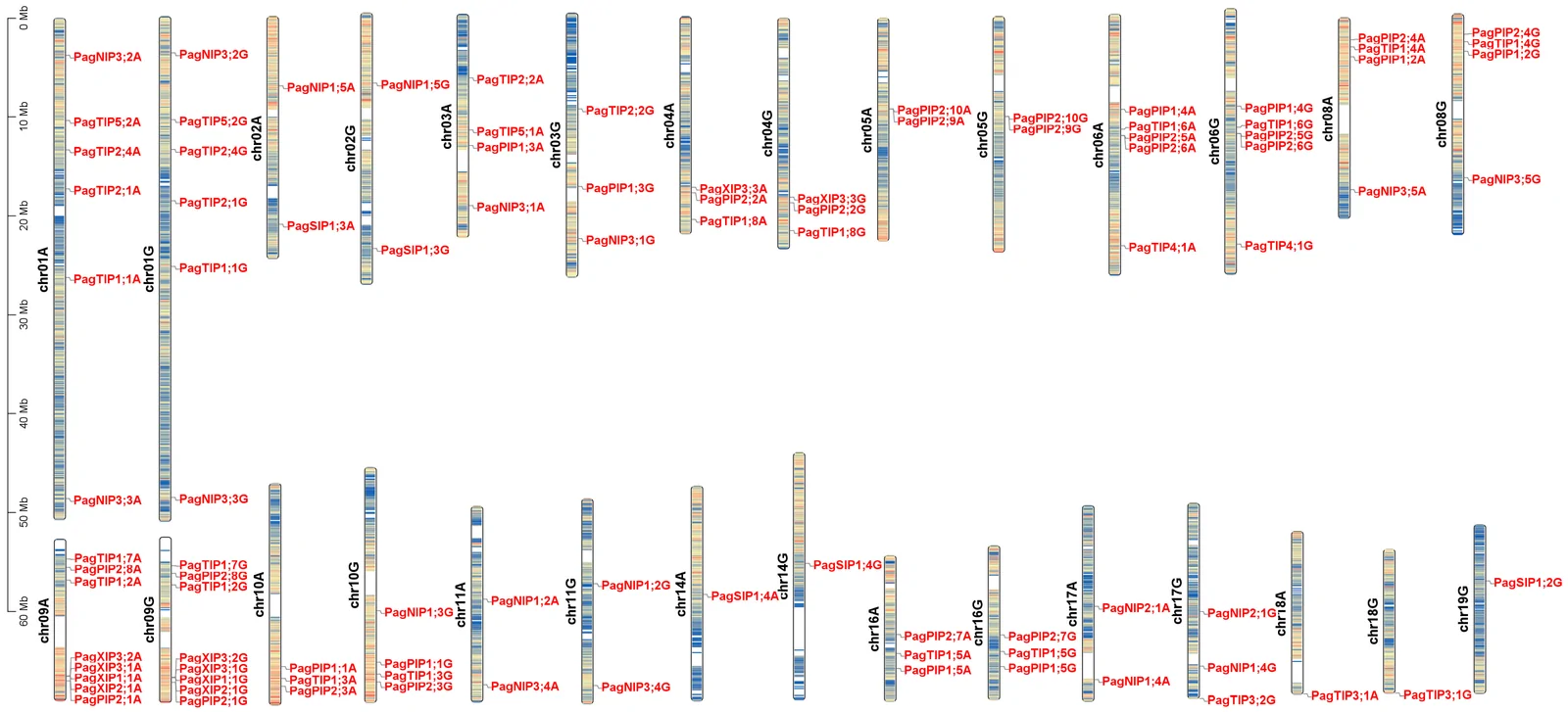
Chromosomal localization map of *PagAQPs* in 84K poplar. Different colors on the chromosomal bars indicate the gene density of different chromosomal regions. Red text labels represent the names of *PagAQP* genes distributed on the chromosomes.

We analyzed the distribution patterns of 94 *PagAQPs* across the two subgenomes of 84K poplar. As shown in Figure 2, all *PagAQPs* were unevenly distributed across 14 and 15 chromosomes of the A and G subgenomes, respectively. chr09A/G, chr01A/G, and chr06A/G contained relatively large numbers of *PagAQPs*, with 8/8, 6/6, and 5/5 genes, respectively, whereas no *PagAQPs* were detected on chr07A/G, chr12A/G, chr13A/G, chr15A/G, or chr19A; the remaining chromosomes contained one to four *PagAQPs*. In addition, most *PagAQPs* exhibited highly consistent chromosomal positions and gene orders between homologous chromosomes of the A and G subgenomes, displaying an allelic-pair distribution pattern. These results indicate that most members of the PagAQP family were symmetrically retained in the two parental subgenomes, whereas only a few members exhibited subgenome-specific retention.

### 2.3. Collinearity and Duplication Analysis of PagAQPs

**Figure 3:**
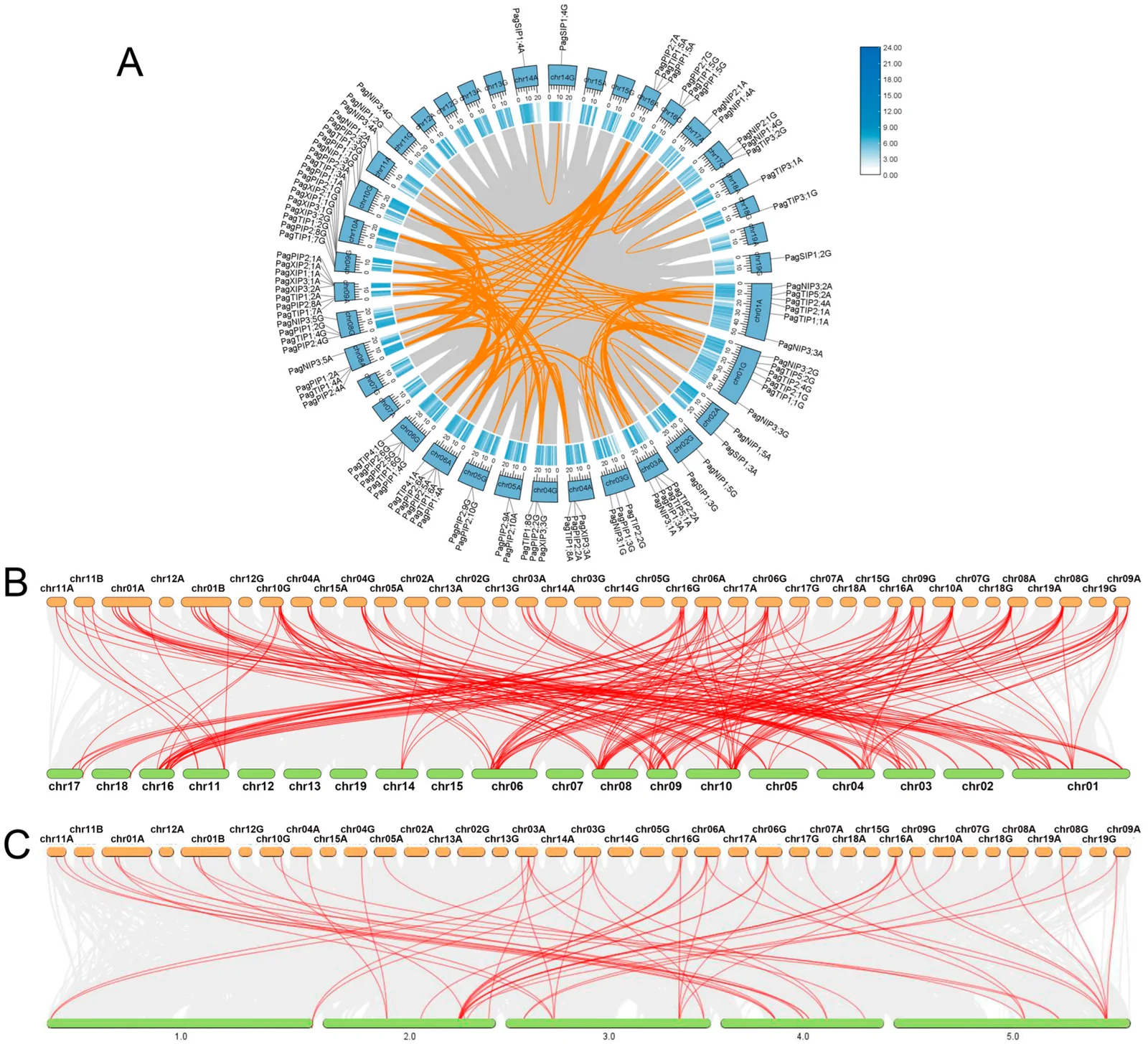
Intraspecific and interspecific synteny analyses of *PagAQPs*. (**A**) Intraspecific collinearity analysis of *PagAQPs*; the gray lines in the innermost circle represent all collinear gene pairs in the 84K poplar genome, and the orange lines indicate collinear *PagAQP* gene pairs. (**B**) Collinearity analysis between 84K poplar and *Populus trichocarpa*. (**C**) Collinearity analysis between 84K poplar and *Arabidopsis thaliana*. The gray lines represent all collinear gene pairs between the 84K poplar genome and other plant genomes, while the red lines denote collinear *AQP* gene pairs. The orange segments represent chromosomes of 84K poplar, while the green segments represent the chromosomes of the reference species (*Populus trichocarpa* in (**B**) and *Arabidopsis thaliana* in (**C**)).

To elucidate the evolutionary and expansion characteristics of the AQP gene family in 84K poplar, we conducted intraspecific and interspecific collinearity analyses and performed Ka/Ks analyses of A/G allelic gene pairs (i.e., collinear homologous gene pairs at corresponding positions on A and G subgenome chromosomes), as well as collinear paralogous gene pairs within each subgenome. Intraspecific collinearity analysis revealed extensive collinearity relationships among *PagAQP* genes across the genome, with 164 collinear gene pairs identified (Figure 3A). Interspecific collinearity analysis identified 208 collinear gene pairs between 84K poplar and the closely related species *Populus trichocarpa* (Figure 3B), but only 56 between 84K poplar and the distantly related species *Arabidopsis thaliana* (Figure 3C), indicating collinearity relationships of *PagAQPs* are more highly conserved between the two *Populus* species.

Ka/Ks analysis identified 38 A/G allelic gene pairs with valid Ka/Ks values, all Ka/Ks ratios were less than 1 and were predominantly distributed between 0 and 0.3 (Table S2), indicating strong purifying selection and evolutionary conservation of the A and G subgenome alleles. Within the A and G subgenomes, 32 and 27 collinear paralogous gene pairs with valid Ka/Ks values were identified, respectively, and all of their Ka/Ks ratios were also less than 1 (Table S3), indicating that retained duplicated PagAQP family members in both subgenomes were subjected to strong purifying selection. Collectively, these analyses revealed extensive collinear retention of *PagAQP* genes within and between the A and G subgenomes, while Ka/Ks ratios below 1 indicated that the retained allelic and within-subgenome paralogous pairs were subjected to purifying selection.

### 2.4. Analysis of Gene Structure, Conserved Domains and Motifs of the PagAQPs Gene Family

**Figure 4:**
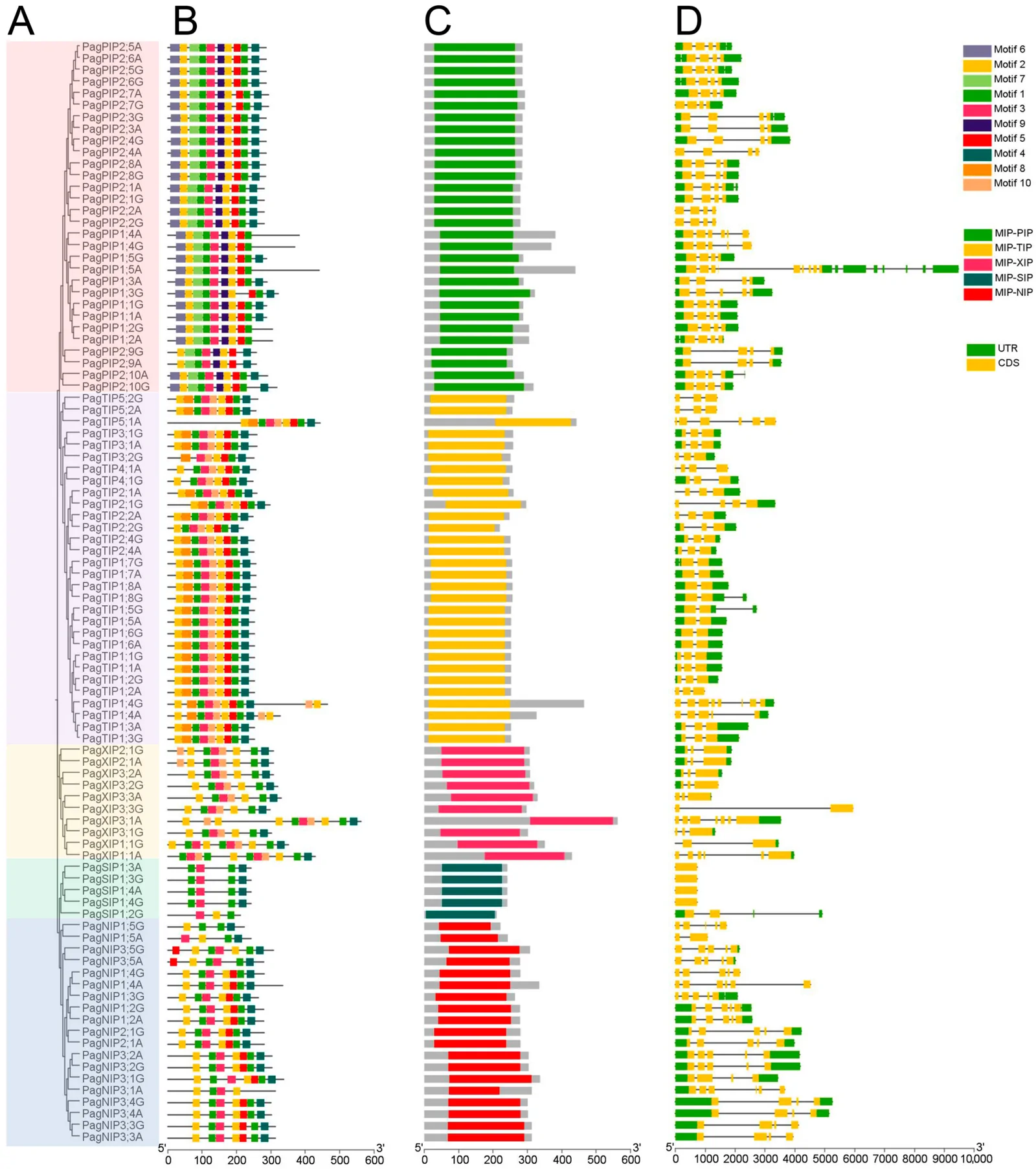
Visualization analysis of conserved structures of PagAQPs. (**A**) Phylogenetic tree of PagAQP proteins. Different background colors represent five subfamilies of 84K poplar: red represents PagPIP subfamily, purple represents PagTIP subfamily, yellow represents PagXIP subfamily, green represents PagSIP subfamily, blue represents PagNIP subfamily. (**B**) Conserved motifs of PagAQPs. Different colors represent different motifs. (**C**) Conserved protein domains of PagAQPs. Different colors represent the representative conserved protein domains of different subfamilies. (**D**) Gene structure of *PagAQPs*. Yellow boxes indicate exons, and green boxes represent UTRs.

Analyses of the conserved motifs, conserved domains, and gene structures of PagAQPs showed that they could be divided into five classical subfamilies (Figure 4A), each exhibiting distinct subfamily-specific features. Regarding conserved motif composition, the number, type, and arrangement of motifs were highly conserved among the same subfamily but differed markedly among different subfamilies (Figure 4B). Each subfamily contained characteristic conserved domains, which was generally consistent with the results of the motif analysis (Figure 4C). Members of the same subfamily exhibited similar exon–intron structural patterns, and the numbers and lengths of exons and introns were highly conserved in most allelic pairs (Figure 4D). Specifically, most PagPIPs contained four exons, whereas PagNIPs predominantly contained five exons, PagTIPs and PagXIPs generally contained two to four exons, and PagSIPs contained one to two exons. These observations indicated that phylogenetically closely related PagAQPs exhibited high consistency in their core motifs, conserved domains, and gene structures, which may confer similar biological functions, whereas structural differences among subfamilies might be associated with their functional divergence.

### 2.5. Protein Structures and Substrate-Specific Residues of PagAQPs

The transport specificity of AQPs for water and small solutes is mainly determined by three highly conserved structural elements: the NPA motifs, Ar/R selectivity filter and Froger sites [4,8]. We compared these structural elements of the PagAQPs, and the results showed that each subfamily exhibited a distinct pattern of conserved specificity (Table S4). The canonical NPA motifs (NPA1 and NPA2) were highly conserved in PagPIPs and PagTIPs, whereas the NPA1 motif varied in PagNIPs, PagSIPs, and PagXIPs, yielding variant motifs including NPS, NPL, NPI, NPV, and NPT, which might be associated with their broad substrate-transport capacities. The ar/R selectivity filter exhibited distinct subfamily-specific characteristics. PagPIPs predominantly contained the conserved F-H-T-R amino acid combination and represented typical aquaporins; PagTIPs mainly contained the characteristic H-I-A-V, H-I-G-R, H-I-A-R, and N-V-G/S-C/S combinations, which might be associated with their functions in water, urea, and ammonia transport; PagXIPs exhibited a relatively conserved V/I/A-V-A-R combinations; PagSIPs exhibited a relatively conserved A/V-V-P-N combinations; and PagNIPs displayed more diverse residue compositions, which might be related to their broad-spectrum substrate-transport capacities. Comparative analysis of Froger’s positions (P1–P5) showed that these positions were relatively conserved in PagPIPs, PagTIPs, PagSIPs, and PagXIPs, but exhibited a higher degree of variation in PagNIPs.

### 2.6. Analysis of Cis-Acting Elements of PagAQPs Promoters

**Figure 5:**
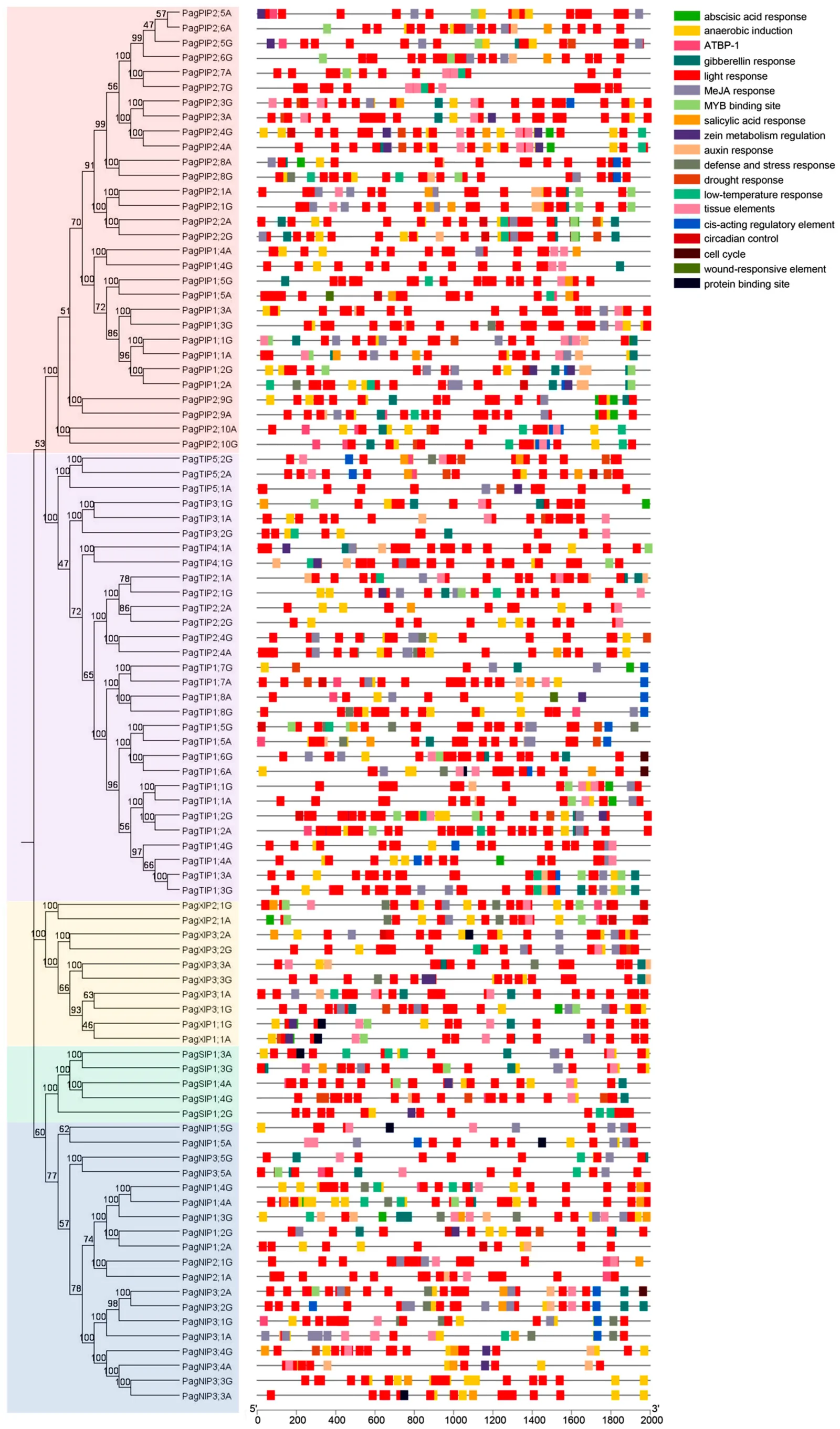
Analysis of cis-acting elements of *PagAQPs*. Different background colors represent five subfamilies of 84K poplar: red represents PagPIP subfamily, purple represents PagTIP subfamily, yellow represents PagXIP subfamily, green represents PagSIP subfamily, blue represents PagNIP subfamily. Different color squares represent different cis-regulatory elements.

To explore the potential transcriptional regulatory mechanisms of PagAQP genes, we identified cis-acting regulatory elements within the 2000 bp sequences upstream of their translation start sites. A total of 19 major types of cis-acting elements were detected (Figure 5) and grouped into eight functional categories: light-responsive, phytohormone-responsive, abiotic-stress-responsive, biotic-stress-responsive, growth- and development-related, transcriptional regulation-related, circadian rhythm-related, and other elements (Table 1 and Table S5). Light-responsive elements were abundant and present in the promoters of all PagAQPs, suggesting that light may represent an important environmental cue regulating PagAQPs transcription. Numerous elements associated with phytohormone and abiotic-stress responses were also identified, supporting the potential involvement of PagAQPs in hormonal signaling and environmental adaptation.

Furthermore, the promoters of PagPIP and PagTIP genes contained the largest numbers of predicted cis-acting elements, including light-responsive elements (e.g., G-box and GT1-motif), abscisic acid-responsive elements (e.g., ABRE), drought-responsive elements (e.g., MBS), auxin-responsive elements (e.g., AuxRR-core and TGA-box), and anaerobic induction elements (e.g., ARE) (Table S5). Further analysis of cis-acting elements in A/G-subgenome allelic pairs revealed differences in element composition and/or abundance between the two allelic copies (Figure 5 and Table S5). For example, PagPIP2;4A contained more GT1-motifs and a unique GARE-motif, whereas PagPIP2;4G contained more AREs and a unique circadian element; similarly, PagTIP1;3G contained more MeJA-responsive, Box 4, and GARE elements, whereas several light-responsive and MYB-related elements were specific to PagTIP1;3A. These promoter differences may contribute to the divergent spatiotemporal and stress-responsive expression of allelic PagAQP genes.

### 2.7. Analysis of Tissue-Specific Expression of PagAQPs in 84K

**Figure 6:**
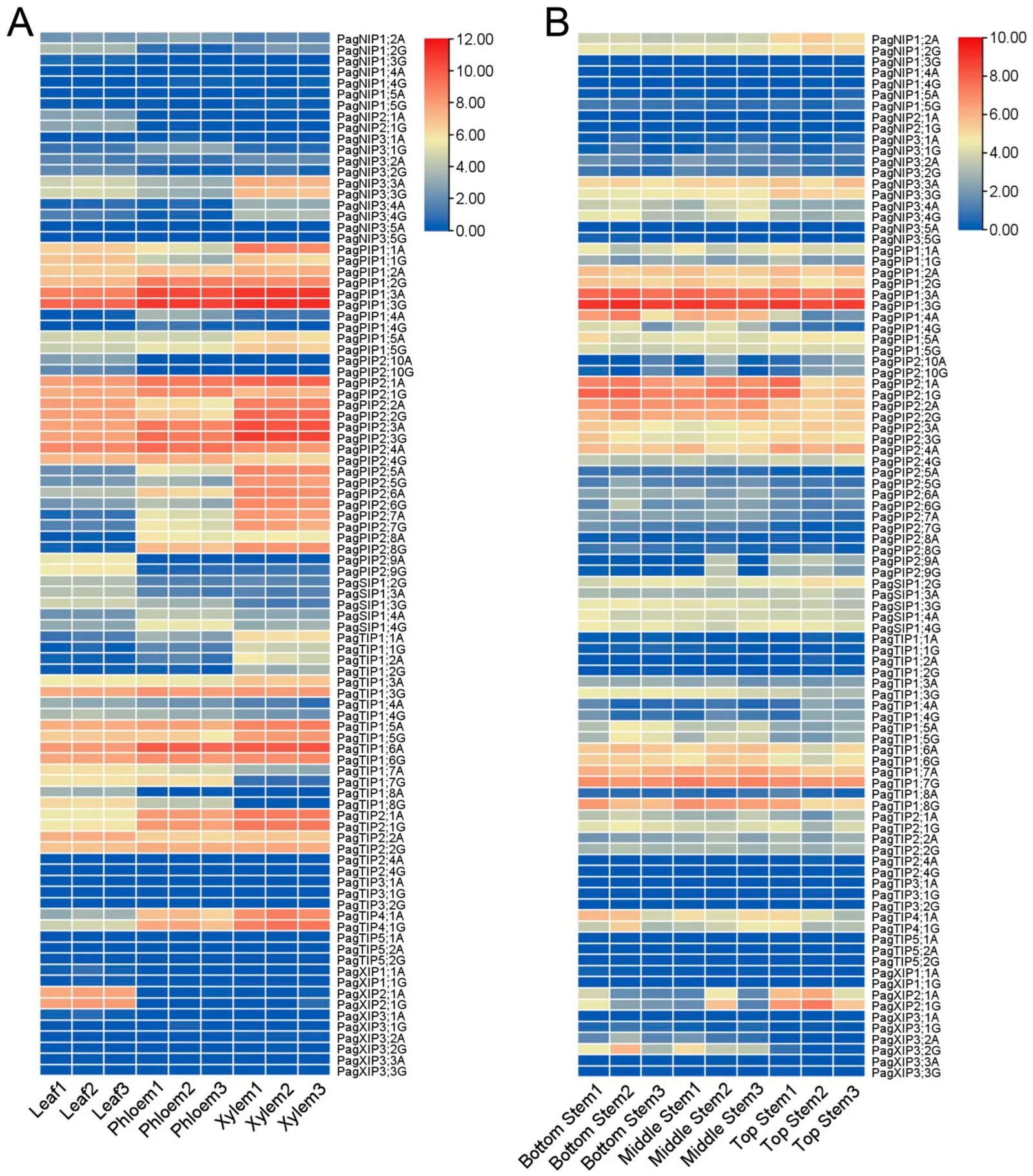
Expression heatmap of *PagAQPs* in different tissues. (**A**) Expression heatmap of *PagAQPs* in leaves, phloem and xylem. (**B**) Expression heatmap of *PagAQPs* in bottom stem, middle stem and top stem.

Transcriptome heatmap analysis showed that *PagAQPs* exhibited distinct tissue-specific expression patterns in 84K poplar (Figure 6A). *PagPIPs* were preferentially expressed in vascular tissues. Among them, *PagPIP1;1*, *PagPIP1;2*, *PagPIP1;3*, *PagPIP2;2*, and *PagPIP2;3* were highly expressed in both xylem and phloem, whereas *PagPIP2;5*, *PagPIP2;6*, and *PagPIP2;7* were predominantly highly expressed in xylem, at lower levels in phloem, and only weakly in leaves, suggesting that these genes may be involved in regulating water transport within vascular tissues. Some *PagTIPs* were highly expressed in leaves, phloem, and xylem, among which *PagTIP1;5*, *PagTIP1;6*, *PagTIP2;1*, and *PagTIP2;2* were predominantly highly expressed in xylem and phloem. By contrast, *PagNIP*, *PagSIP*, and *PagXIP* genes generally exhibited very low transcript abundance across the tissues examined, with *PagNIP3;3* showing moderate expression in xylem and *PagXIP2;1* showing relatively high expression in leaves (Figure 6A). In addition, *PagAQPs* exhibited higher transcript abundance in older stems than in young stems (Figure 6B). Notably, some *PagAQP* allelic pairs exhibited different expression patterns. For example, the A-subgenome copies of *PagPIP1;1*, *PagPIP2;1*, and *PagPIP2;4* showed higher expression levels in the xylem than their corresponding G-subgenome alleles, whereas the A-subgenome copies of *PagPIP2;8* and *PagTIP1;3* showed lower expression levels than their corresponding G-subgenome alleles. Nevertheless, most A/G allelic pairs of *PagAQPs* exhibited broadly similar expression patterns. These results suggest that *PagAQPs* from different subfamilies may have undergone functional divergence during evolution. In particular, some *PagPIP* and *PagTIP* members were highly expressed in vascular tissues, suggesting that they may participate in water transport and water-homeostasis regulation in 84K poplar.

### 2.8. Responses of PagAQPs to Abiotic Stresses in 84K Poplar

**Figure 7:**
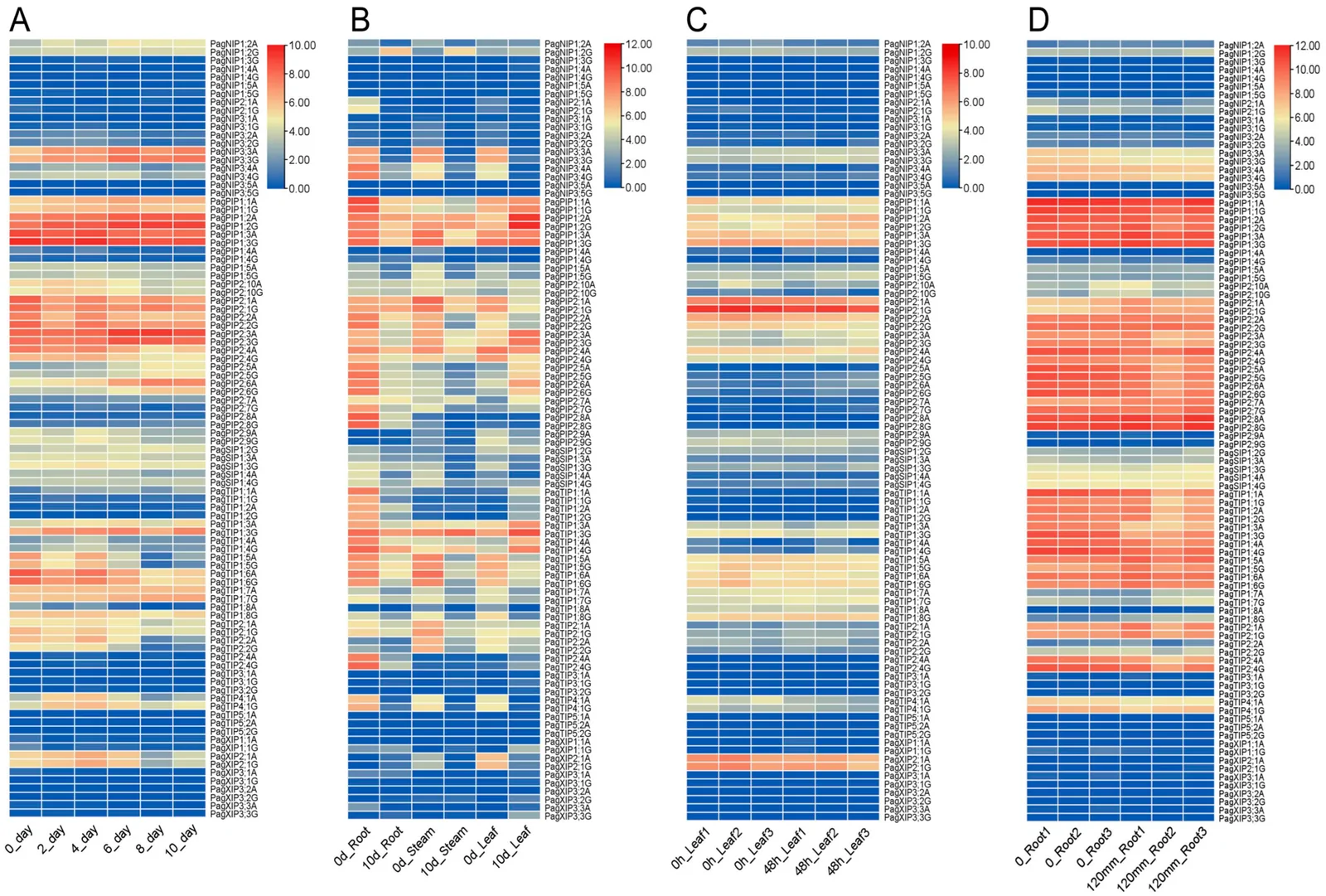
Expression profiles of *PagAQPs* under different abiotic stresses. (**A**) Expression heatmap of *PagAQPs* in leaves under nitrogen stress. (**B**) Expression heatmap of *PagAQPs* in roots, stems and leaves after 10 days of drought treatment. (**C**) Expression heatmap of *PagAQPs* in leaves after treatment with 100 mM NaCl solution. (**D**) Expression heatmap of *PagAQPs* in roots after treatment with 120 mM NaCl solution.

Water, nitrogen, and salt are important environmental factors affecting plant growth and development [34,35]. Accordingly, we analyzed the transcriptional responses of *PagAQPs* to nitrogen, drought, and salt stresses. After four days of nitrogen stress, the transcript abundance of *PagPIP2;3A/G* and *PagPIP2;6A/G* began to increase, whereas those of genes such as *PagTIP1;5A/G* and *PagTIP2;2A/G* began to decrease. Among *PagNIPs*, *PagSIPs*, and *PagXIPs*, only *PagXIP2;1A/G* initially exhibited increased expression under nitrogen stress, followed by a decrease after four days, whereas the remaining family members were largely unaffected by nitrogen stress (Figure 7A).

After 10 days of drought stress, most *PagPIPs* and *PagTIPs* were downregulated primarily in roots and stems, whereas *PagPIP1;2A/G*, *PagPIP2;3A/G*, *PagPIP2;5A/G*, and *PagTIP1;4A/G* were upregulated in leaves, indicating that these genes were specifically responsive to drought stress (Figure 7B). These results were consistent with the abundance of abscisic acid, MeJA, and drought responsive elements in the promoter regions of *PagPIPs* and *PagTIPs* (Table 1). Under salt stress, the overall transcript abundance of *PagAQPs* remained largely unchanged in both leaves and roots, with only a few genes showing slight fluctuations (Figure 7C,D). Notably, under nitrogen and drought treatments, most allelic pairs of *PagAQPs* exhibited broadly similar expression profiles, whereas some pairs showed differences in their estimated expression levels. For example, *PagPIP2;4A* and *PagPIP2;6A* showed higher expression levels than their corresponding G-subgenome alleles, whereas *PagTIP1;3G*, *PagTIP1;8G*, and *PagTIP4;1G* showed higher expression levels than their corresponding A-subgenome alleles. These observations are generally consistent with previous reports [33]. Overall, *PagPIPs* and *PagTIPs* exhibited relatively pronounced changes in transcript abundance under nitrogen deficiency and drought, suggesting that some of these genes may participate in water-homeostasis regulation and nutrient transport in 84K poplar under these conditions.

### 2.9. qRT-PCR Analysis of the Expression of 12 PagAQP Genes Before and After Water Withholding

**Figure 8:**
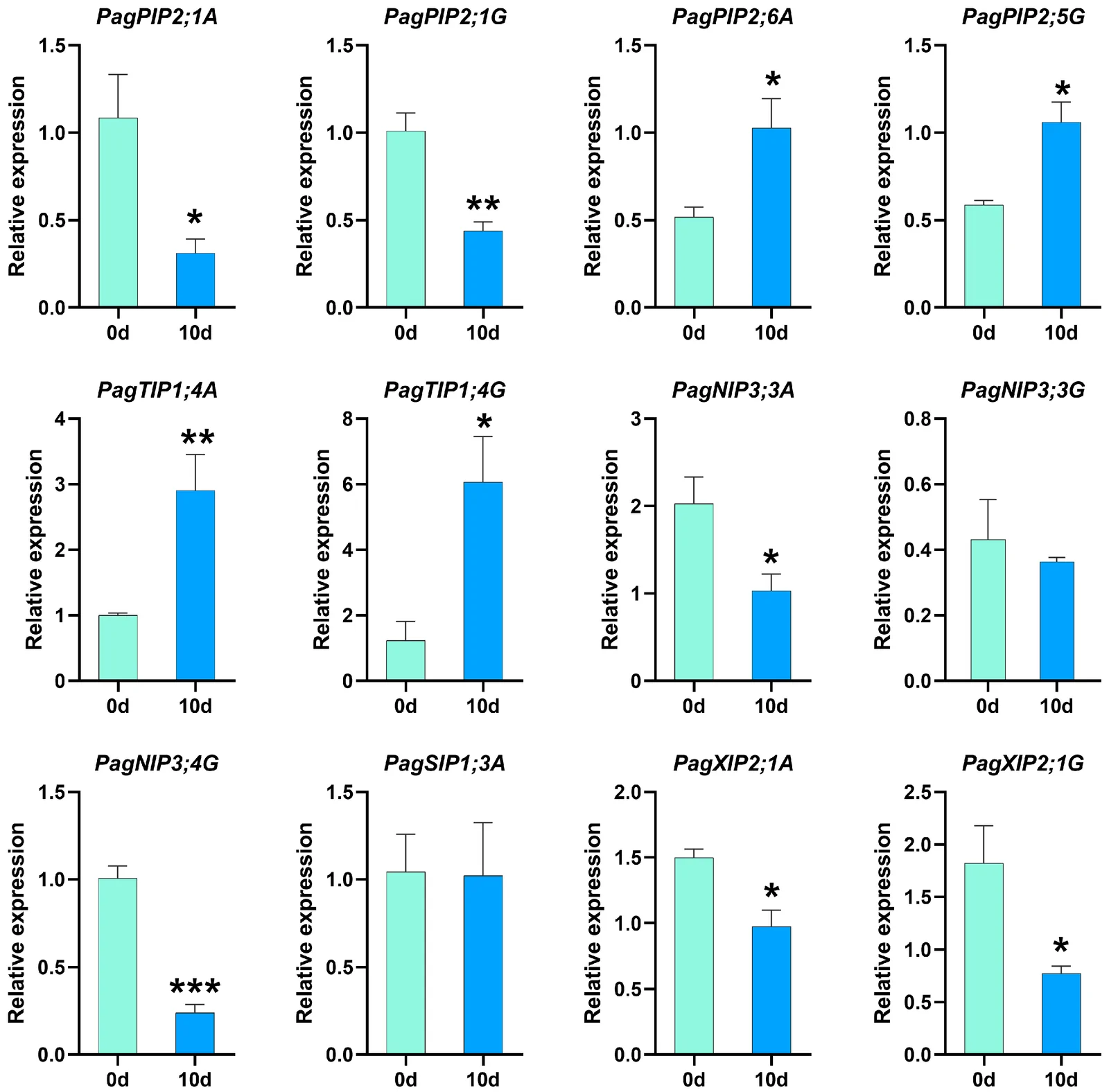
Expression analysis of 12 *PagAQPs* in leaves of 84K poplar before (day 0) and after 10 days of water withholding treatment, measured by qRT-PCR. Asterisks indicate significance levels: * *p* < 0.05, ** *p* < 0.01, *** *p* < 0.001, *t* test.

Transcriptomic analysis revealed that *PagAQPs* exhibited diverse transcriptional responses to drought stress (Figure 7B). To further examine these expression patterns, we measured the relative expression levels of 12 *PagAQPs* in leaves before and after 10 days of water withholding by qRT-PCR. The results showed that *PagPIP2;6A*, *PagPIP2;5G*, and *PagTIP1;4A/G* were upregulated, whereas the remaining genes were downregulated to varying degrees (Figure 8). These expression trends were generally consistent with the transcriptomic results (Figure 7B). Collectively, these observations suggest that changes in the expression of distinct PagAQP family members may contribute to drought stress responses in 84K poplar.

### 2.10. Phenotypic and Physiological Responses of PagPIP2;6A-Overexpressing Transgenic Poplar to Water Withholding

**Figure 9:**
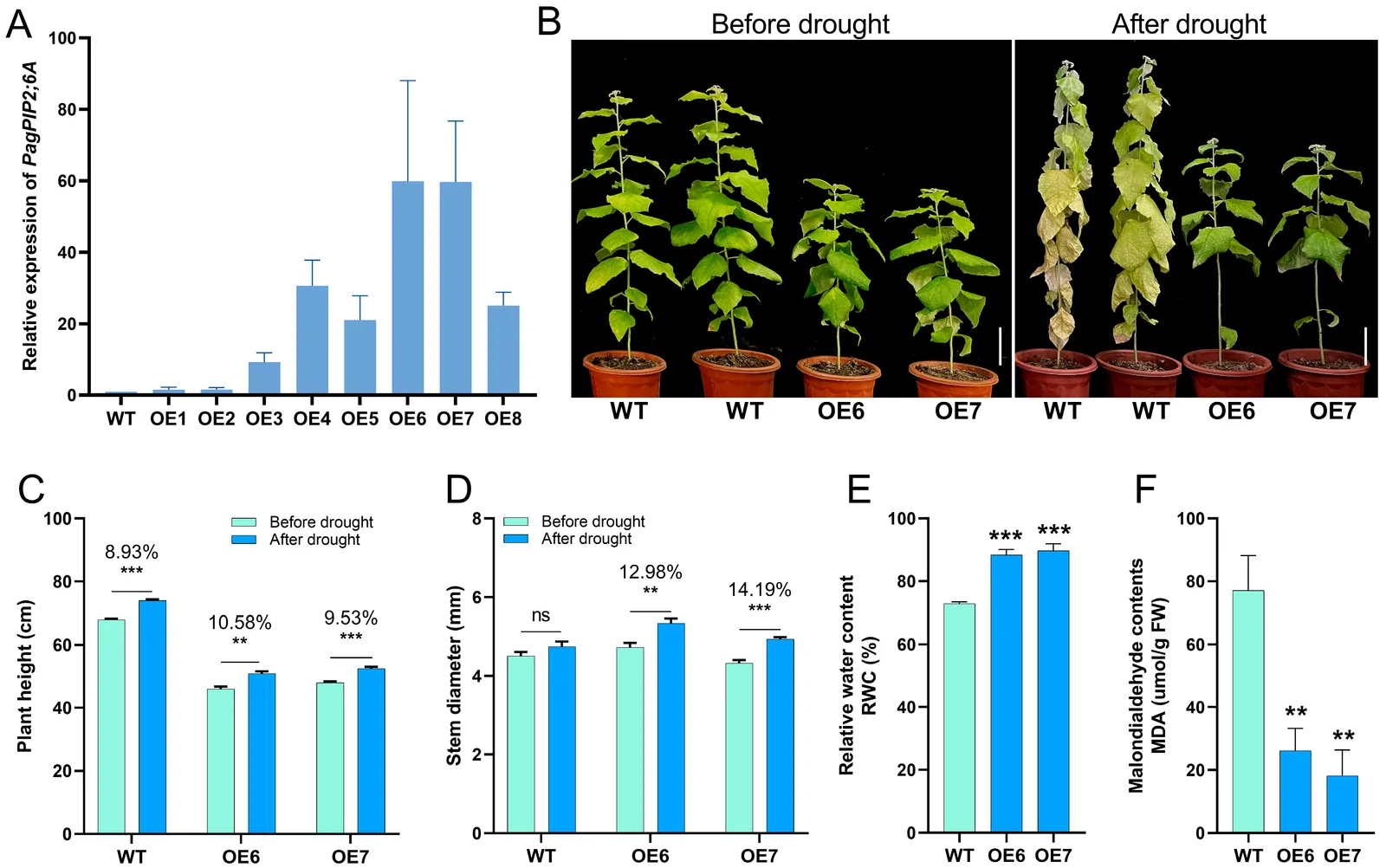
Phenotypic and physiological responses of *PagPIP2;6A*-overexpressing plants to drought treatment. Two-month-old WT and OE plants were subjected to drought treatment by withholding water for 15 days. (**A**) qRT-PCR analysis of *PagPIP2;6A* expression in transgenic lines of 84K poplar. (**B**) Phenotypes of WT and OE lines OE6 and OE7 before and after drought treatment. (**C**,**D**) Statistical analysis of plant height (**C**) and stem diameter (**D**) before and after drought treatment. (**E**,**F**) Measurements of RWC (**E**) and MDA content (**F**) in upper leaves after drought treatment. Data are presented as mean ± SD, *n* = 3. Three biological replicates (independent plants) for WT or each transgenic line were analysed. Asterisks indicate significance levels: ** *p* < 0.01, *** *p* < 0.001, ns indicates no significant difference, *t* test.

Transcriptome profiling indicated that the transcript abundance of *PagPIP2;6A* increased under drought treatment (Figure 7B). qRT-PCR analysis also detected higher relative expression levels after 10 days of water withholding than before treatment (Figure 8), showing a trend consistent with the transcriptomic data. Based on these expression patterns and the established role of PIPs in transmembrane water transport, *PagPIP2;6A* was selected for further analysis. To explore its potential biological effects, we generated 35S:*PagPIP2;6A* transgenic lines of 84K poplar. Among the transgenic lines examined, OE6 and OE7 exhibited the highest *PagPIP2;6A* expression levels and were selected for subsequent phenotypic and physiological analyses (Figure 9A).

Two-month-old wild-type (WT) and *PagPIP2;6A*-overexpressing (OE) plants of 84K poplar were subjected to 15 days of water withholding treatment. Before treatment, OE6 and OE7 were both significantly shorter than WT, indicating that constitutive *PagPIP2;6A* overexpression was associated with reduced plant height (Figure 9B). After drought treatment, the leaves of WT plants showed pronounced yellowing and wilting, whereas the leaves of OE6 and OE7 were less severely damaged and retained the capacity for growth (Figure 9B). Analysis of plant height and stem diameter showed that, relative to pretreatment values, plant height increased by 10.58% and 9.53% in OE6 and OE7, respectively, with both percentage increases exceeding that observed in WT (Figure 9C); stem diameter showed no significant change in WT but increased significantly by 12.98% and 14.19% in OE6 and OE7, respectively (Figure 9D). Further measurements of relative water content (RWC) and malondialdehyde (MDA) content in leaves revealed that transgenic lines had significantly higher RWC (Figure 9E) and significantly lower MDA levels (Figure 9F) than WT after water withholding treatment. Collectively, these observations suggest that constitutive overexpression of *PagPIP2;6A* is associated with reduced vegetative growth and altered physiological responses to drought treatment. Under the tested conditions, the overexpression lines were shorter than WT before treatment and exhibited higher leaf relative water content and lower MDA content after drought treatment.

## 3. Discussion

Plant aquaporins are essential channel proteins that mediate the transmembrane transport of water and small neutral solutes in plants, and are extensively involved in pivotal physiological processes including water homeostasis, growth and development, as well as stress adaptation [36,37]. In this study, a total of 94 *PagAQPs* were identified in the whole genome of 84K poplar and classified into five classic subfamilies (Figure 1). This classification pattern was consistent with those reported in perennial woody plants such as *Populus trichocarpa*, *Hevea brasiliensis*, and *Camellia sinensis*, supporting the overall evolutionary conservation of the AQP family in angiosperms [14,15,38]. Although 84K poplar harbors more *PagAQP* genes than *Populus trichocarpa* (55) [15], its A and G subgenomes contain only 46 and 48 *PagAQP* genes, respectively (Table S1), both fewer than in *Populus trichocarpa*. Thus, the larger overall PagAQP gene family in 84K poplar primarily reflects the combination of two independently evolved parental gene complements through hybridization rather than lineage-specific expansion after hybridization.

Plant PIP and TIP subfamilies predominantly localize to the plasma membrane and tonoplast, and function to facilitate transcellular water transport and the exchange of water and small solutes between the cytosol and vacuole, respectively [39]. Numerous studies have shown that PIP and TIP are the two largest subfamilies of plant AQPs. For example, the 55 AQPs in *Populus trichocarpa* include 15 PIPs and 17 TIPs, the 51 AQPs in *Hevea brasiliensis* include 15 PIPs and 17 TIPs, and the 66 AQPs in *Glycine max* include 22 PIPs and 23 TIPs [13,15,38]. In the present study, PagPIP and PagTIP were the two largest subfamilies of the PagAQP gene family in 84K poplar, with both being relatively balanced between the A and G subgenomes, each exhibiting a 15/15 distribution, broadly consistent with findings in other plant species. This balanced retention across the two subgenomes might provide a molecular basis for the rapid growth, efficient water transport, and long-distance vascular transport of hybrid poplar 84K [30,31]. In addition, the haplotype genome of *Populus trichocarpa* contains 17 TIPs, 11 NIPs, 6 XIPs, and 6 SIPs, respectively, whereas the corresponding numbers in the A/G subgenomes of 84K poplar are 15/15, 9/10, 5/5, and 2/3, respectively (Table S1), indicating that these four subfamilies were lost to varying degrees during divergence of the A and G subgenomes. Overall, the AQP subfamilies in 84K poplar exhibit subfamily-specific retention patterns: PIP and TIP members are relatively conserved, whereas the NIP, XIP, and SIP subfamilies show varying degrees of contraction and slight subgenomic asymmetry. These findings indicate that the evolution of the PagAQP gene family in 84K poplar does not simply reflect the additive combination of the two parental gene complements but may have been jointly shaped by functional constraints, selective retention, and gene loss.

The chromosome mapping revealed that *PagAQPs* were unevenly distributed across 29 chromosomes (Figure 2), a pattern also widely reported for *AQP* genes in other plant species [40]. Furthermore, the majority of independently retained duplicated genes within the A and G subgenomes occupied corresponding positions and displayed conserved gene order on homologous chromosomes, forming an allelic pair distribution pattern (Figure 2), implying that no large-scale loss of *PagAQP* genes occurred during subgenome divergence, thereby providing an evolutionary basis for the functional integrity and coordinated functions of the PagAQP gene family in hybrid poplar 84K. A similar pattern of highly symmetrical AQP retention has been reported between the A and D subgenomes of allotetraploid *Gossypium hirsutum* [41]. The highly conserved collinearity between 84K and *Populus trichocarpa* (Figure 3B) was consistent with their close phylogenetic relationship within the *Salicaceae* family, whereas the lower level of collinearity with *Arabidopsis thaliana* (Figure 3C) likely reflects deeper evolutionary divergence accompanied by accumulated chromosomal rearrangements and gene turnover, rather than directly demonstrating specific gene-loss events [42,43]. Nevertheless, the collinear gene pairs retained between these distantly related taxa indicate that a subset of AQP loci has remained evolutionarily conserved, consistent with the fundamental roles of AQPs in water and solute transport [44,45,46].

Ka/Ks analysis showed that both allelic and paralogous gene pairs were predominantly under purifying selection, with most Ka/Ks ratios falling between 0 and 0.3 (Tables S2 and S3). This evolutionary pattern has also been reported in the AQP families of the allopolyploid species, such as *Gossypium hirsutum*, *Triticum aestivum*, and *Brassica napus*, etc. [41,47,48], indicating that the key functional domains in the coding regions of homologous *PagAQPs* contributed by the two parental subgenomes were under strong selective constraint and supporting, to some extent, the functional conservation of the PagAQP gene family. However, although homologous genes derived from different subgenomes are highly conserved in their coding regions, sequence variation in their upstream regulatory regions may lead to differences in spatial and temporal expression patterns and stress responses, resulting in functional divergence within the gene family [41,47]. Previous studies on the AQP gene family in *Triticum aestivum* showed that duplicated genes retained functional conservation in their coding regions under purifying selection while exhibiting significant expression divergence among different organs and developmental stages and under water stress [47]. In the present study, the promoter regions of homologous *PagAQPs* derived from the A and G subgenomes of 84K poplar contained numerous distinct *cis*-regulatory elements (Figure 5, Table 1). These findings suggested that the *PagAQP* gene family of 84K poplar might similarly maintain a high degree of functional conservation through purifying selection while achieving functional divergence through variation in regulatory regions, reflecting an evolutionary strategy that enhanced environmental adaptability while retaining the core physiological functions inherited from both parents.

Analyses of gene structure, conserved motifs, and domains showed that the characteristic domains and motifs were highly conserved among members of the same subfamily but differed markedly among subfamilies, corresponding closely to their phylogenetic relationships (Figure 4). This structural conservation within subfamilies and divergence among subfamilies might provide a structural basis for the functional divergence of PagAQPs. The substrate transport specificity and channel functions of plant AQPs are determined primarily by the combined effects of three conserved structural elements: the NPA motifs, the ar/R selectivity filter, and Froger positions [49,50]. In the present study, these three structural elements exhibited clear subfamily specificity among PagAQPs (Table S4). All three elements were highly conserved in PagPIPs, as also reported for PIPs in other higher plants [51,52], suggesting that these proteins primarily mediate transmembrane water transport. PagTIPs contained highly conserved NPA motifs but diverse ar/R residue combinations, indicating that they might transport multiple substrates, including water, urea, and ammonia, as supported by the reported NH_3_ transport activity of *AtTIP2;1* and *AtTIP2;3* in *Arabidopsis thaliana* [53]. PagNIPs, PagSIPs, and PagXIPs exhibited diverse variants at the NPA1 site, as also reported in *Populus trichocarpa*, *Camellia sinensis*, and *Kandelia obovata*, suggesting that these proteins might also mediate the broad spectrum transport of small molecules such as boron, silicon, and glycerol [14,15,52].

Previous studies have demonstrated that in crops, regulating the expression of rice *OsPIP1;3* can significantly enhance photosynthesis and disease resistance [54], while *OsNIP3;1* promoted boron absorption and transport in rice [44]. Modulation of *TaPIP2;10* phosphorylation in wheat markedly improved photosynthesis and defense capabilities [45], whereas overexpression of *ZmPIP1;1* in maize significantly enhanced photosynthesis and drought resistance [46]. In woody plants, overexpression of *PtPIP1;1* and *PtdPIP2;5* similarly enhanced photosynthesis and drought resistance in poplar [27,55]. These findings indicated that plant aquaporins participate in regulating light and abiotic/biotic stresses. Cis-acting element analysis revealed that the promoters of *PagAQP* genes contained predicted elements associated with light, ABA, MeJA, drought, anaerobic induction, and gibberellin responses (Figure 5). These findings suggest that *PagAQPs* expression may be influenced by environmental and hormonal signals, providing clues to potential regulatory mechanisms underlying their roles in plant growth, development, and stress responses. However, the regulatory functions of these predicted elements require experimental validation.

Tissue specific expression analysis showed that *PagPIPs* and *PagTIPs* were preferentially expressed at high levels in vascular tissues, including xylem and phloem, whereas *PagNIPs*, *PagSIPs*, and *PagXIPs* generally exhibited low expression levels (Figure 6A), suggesting that *PagPIPs* and *PagTIPs* might be key components of water and nutrient transport in 84K poplar. In addition, most allelic pairs exhibited similar expression patterns, supporting a previous report that allelic expression in 84K poplar showed no transcriptional bias between the two subgenomes [33]. Previous studies have demonstrated that *PtPIP2;7*, *PtPIP2;8*, and *PtTIP4;1* were highly expressed in xylem, roots, and phloem under drought stress [56], whereas the expression of *OsPIP1;1*, *OsPIP2;3*, *OsPIP2;4*, *OsPIP2;5*, *OsTIP1;1*, *OsTIP1;2*, and *OsTIP2;2* increased significantly under sufficient nitrogen supply [57,58]. In this study, many *PagPIP* and *PagTIP* members showed expression changes under drought and nitrogen treatments (Figure 7A,B), and their promoters contained predicted cis-regulatory elements, including ABRE, MBS, AuxRR-core, TGA-box, and ARE (Table S5). These observations suggest that the predicted cis-regulatory elements may contribute to the regulation of *PagPIP* and *PagTIP* expression under these treatment conditions. qRT-PCR analysis further showed increased expression of *PagPIP2;6A*, *PagPIP2;5G*, and *PagTIP1;4A/G* after water withholding relative to pretreatment expression levels (Figure 8), with trends broadly consistent with those observed in the drought transcriptomic data. Together, these findings suggest that members of the PagPIP and PagTIP subfamilies may participate in responses to water and nitrogen availability, potentially through their roles in the transport of water and small solutes.

As major aquaporins, PIPs regulate plant growth by modulating cellular water homeostasis, turgor pressure balance, root hydraulic conductivity, and cell elongation [25]. Previous studies have demonstrated that constitutive overexpression of *SikPIP1;5A* in tomato significantly repressed plant growth under normal growth conditions, whereas it remarkably enhanced cold tolerance of transgenic plants under cold stress [59]. Overexpression of *OsPIP1;1* in *Oryza sativa* drastically inhibited root and leaf growth, yet improved plant salt tolerance and germination capacity [60]. In this study, constitutive overexpression of *PagPIP2;6A* resulted in shorter plant height compared with the WT (Figure 9B,C), while the overexpression lines displayed higher leaf RWC (Figure 9E) and lower MDA content (Figure 9F) after drought treatment. Together with previous reports, these observations suggest that sustained PIP overexpression may impose a vegetative growth penalty while altering physiological responses to environmental stress. However, in this study, because initial plant size differed and soil water status was not matched among genotypes, the present results do not conclusively establish a trade-off between intrinsic drought tolerance and growth.

In poplar, *PtdPIP2;5*-overexpressing lines significantly enhance plant drought tolerance by promoting root hydraulic conductance and improving plant water flux [55], whereas overexpression of *PtPIP2;8* significantly reduces plant drought tolerance [61], suggesting that different PIP family members may have distinct functions. In this study, *PagPIP2;6A* OE lines maintained higher leaf RWC (Figure 9E) and lower MDA (Figure 9F) after drought treatment, which partly implied that overexpressed *PagPIP2;6A* may preserve plant growth capacity under drought by alleviating leaf water loss and membrane lipid peroxidation damage. Nevertheless, physiological parameters such as cell length, stomatal conductance, transpiration rate, leaf water potential, and root and stem hydraulic conductance were not measured in the transgenic plants in this study. Therefore, the physiological basis of the reduced plant height and altered responses to drought stress associated with *PagPIP2;6A* overexpression remains unclear. Moreover, although OE6 and OE7 exhibited similar growth and drought response patterns, evidence from these two overexpression lines provides only preliminary support for the potential function of *PagPIP2;6A*. Further validation using rigorously controlled water conditions, additional independent transgenic lines, and complementary loss-of-function materials is therefore required.

Furthermore, under the drought treatment conditions of this study, the *PagPIP2;6A* overexpression lines displayed higher leaf RWC and lower MDA content than WT, suggesting relatively greater leaf water retention and lower membrane lipid peroxidation, while maintaining some capacity for growth. These observations imply that *PagPIP2;6A* may have potential value for the genetic improvement of drought responses in forest trees, although further validation under rigorously controlled water conditions is required. However, constitutive expression of *PagPIP2;6A* driven by the 35S promoter may impose a growth penalty. If future studies further confirm its beneficial effects under water deficit, promoters inducible by drought or ABA could be considered to upregulate *PagPIP2;6A* primarily during water deficit while maintaining relatively low expression under adequate water supply. This strategy may help balance favorable responses to water deficit with normal vegetative growth.

## 4. Materials and Methods

### 4.1. Identification of PagAQPs Gene Family Members in 84K Poplar

To identify AQP gene family members in the whole genome of 84K poplar [33], the amino acid sequences of AQPs from previously reported *Arabidopsis thaliana* [11] and *Populus trichocarpa* [15] were employed as query sequences. A BLASTp search was conducted against the 84K poplar genome protein database (https://ngdc.cncb.ac.cn/gwh/Assembly/26017/show, accessed on 2 March 2026) using the TBtools-II software (v2.485) with an E-value <1 × 10^−10^ [62]. The Hidden Markov Model (HMM) profile of the MIP domain (PF00230) was obtained from the Pfam database (http://pfam.xfam.org/, accessed on 2 March 2026), and used with HMMER 3.3 to identify candidate AQP family members in the 84K genome (E-value ≤0.01). The conserved domains of the candidate protein sequences were subsequently verified using three online bioinformatics tools: the InterPro database (https://www.ebi.ac.uk/interpro/, accessed on 2 March 2026) [63], SMART (http://smart.embl.de/, accessed on 2 March 2026) [64], and CDD (https://www.ncbi.nlm.nih.gov/Structure/bwrpsb/bwrpsb.cgi, accessed on 2 March 2026) [65]. Candidates obtained from the two searches were merged and verified using InterPro, SMART, and CDD. An E-value threshold of ≤0.01 was applied to CDD, whereas the default parameters were used for InterPro and SMART. For multiple protein isoforms derived from the same annotated locus, only the longest complete isoform was retained. Proteins showing obvious truncation, anomalous domain architecture, or lacking a complete MIP domain were excluded. A total of 94 *PagAQP* gene family members were ultimately identified (Table S1).

### 4.2. Characterization of PagAQP Proteins of 84K Poplar

The physicochemical properties of the identified PagAQP proteins, including amino acid length, molecular weight, theoretical isoelectric point, instability index, aliphatic index, and grand average of hydropathicity, were predicted using the online tool ProtParam (https://web.expasy.org/protparam/, accessed on 2 March 2026, parameters were set to default) on the ExPASy website [66]. Subcellular localization of the PagAQP proteins was predicted using the online tool WoLF PSORT (https://wolfpsort.hgc.jp/, accessed on 2 March 2026, parameters were set to default). The number of transmembrane domains of the PagAQP proteins were analyzed using the online tool TMHMM-2.0 (https://services.healthtech.dtu.dk/services/TMHMM-2.0/, accessed on 2 March 2026, parameters were set to default).

### 4.3. Gene Structure, Conserved Motifs and Domain Analysis of PagAQPs

The conserved motifs of the *PagAQPs* were identified using the online tool MEME (https://meme-suite.org/meme/tools/meme, accessed on 2 March 2026) [67]. Conserved domains were analyzed using the Batch-CD-Search function on the NCBI website (https://www.ncbi.nlm.nih.gov/Structure/bwrpsb/bwrpsb.cgi, accessed on 2 March 2026, E-value ≤ 0.01). The gene structures, characteristic motifs, and domains were visualized using the visualization module of TBtools-II (v2.485).

### 4.4. Phylogenetic Analysis and Classification of AQPs

To clarify the phylogenetic evolutionary relationships of the AQP gene family in hybrid 84K poplar, multiple sequence alignments of AQP protein sequences from 84K poplar, *Arabidopsis thaliana*, and *Populus trichocarpa* were performed using the ClustalW program embedded in MEGA 12.0.14 software with the default settings [68]. The gap-opening and gap-extension penalties were set to 10.0 and 0.10 for pairwise alignment and 10.0 and 0.20 for multiple alignment, respectively, with all other parameters set to default. The phylogenetic tree of PagAQPs was constructed using the Neighbor-Joining (NJ) method with amino acid *p*-distances in MEGA 12.0.14, with all other parameters set to default. Bootstrap analysis was performed with 1000 replicates. The phylogenetic tree was further visualized using iTOL (v6, https://itol.embl.de/, accessed on 4 March 2026) [69]. The *PagAQPs* of 84K poplar were named according to the sequence homology and phylogenetic relationships with AQP members from *Arabidopsis thaliana* and *Populus trichocarpa*.

### 4.5. Chromosomal Localization and Collinearity Analysis of PagAQP Gene Family Members

Chromosomal location information of all *PagAQPs* was extracted from the 84K poplar genome and the GFF3 annotation file. Intraspecific and interspecific collinearity analysis was performed using MCScanX of TBtools-II (v2.485) with an E-value <1 × 10^−10^. The chromosomal distribution map and collinearity analysis plots of *PagAQP* genes were generated using TBtools-II (v2.485). The synonymous substitution rate (Ks) and non-synonymous substitution rate (Ka) of collinear gene pairs were calculated via the Ka/Ks Calculator module in TBtools-II (v2.485) with default parameters, and the selection pressure was evaluated by the Ka/Ks ratio (a ratio less than 1 indicates purifying selection, equal to 1 indicates neutral evolution, and greater than 1 indicates positive selection).

### 4.6. Analysis of Substrate Selectivity-Related Sites of PagAQP Proteins

To analyze the substrate transport specificity of PagAQP proteins, sequence alignment and characterization were performed on the NPA motifs (Asn-Pro-Ala), the aromatic/arginine (Ar/R) selectivity filter, and the Froger sites (P1–P5) of PagAQP proteins based on the reported protein sequence structure of KoPIP2;1 from *Kandelia obovata* [52]. Based on the amino acid residue composition of the aforementioned sites within each subfamily, the potential transport properties for water molecules or small molecule solutes were inferred, and differences between allelic pairs were compared.

### 4.7. Analysis of Cis-Acting Elements in the Promoters of PagAQPs

The 2000 bp nucleotide sequence upstream of the translation start site of each *PagAQP* gene was extracted. The cis-acting elements within the promoter sequences were predicted using the PlantCARE online database (https://bioinformatics.psb.ugent.be/webtools/plantcare/html/, accessed on 2 March 2026, default parameters), and the results were subsequently statistically analyzed and categorized.

### 4.8. Transcriptomic Analysis of Tissue-Specific Expression and Abiotic Stress Response of PagAQPs

Six publicly available RNA-seq expression matrices of 84K poplar were downloaded from the NGDC database (https://ngdc.cncb.ac.cn/ppgr/, accessed on 15 March 2026). The downloaded data were provided as normalized transcripts per million (TPM) values, and log_2_(TPM + 1)-transformed for expression heatmap of *PagAQPs* visualization using TBtools-II software. The expression patterns of *PagAQPs* were analyzed in different tissues and organs (leaf, phloem, xylem; BioProject accession: PRJNA526157), different stem segments (basal stem, middle stem, top stem; BioProject accession: PRJNA775903), nitrogen stress (leaf; BioProject accession: PRJNA629424), drought stress (root, stem, leaf; BioProject accession: PRJCA003153), and salt stress (leaf and root; BioProject accessions: PRJNA803823 and PRJNA796558).

### 4.9. Plant Materials and Growth Conditions

Wild-type and transgenic plants of 84K poplar were cultivated in a greenhouse at 25 °C with a photoperiod of 16 h light/8 h dark and a light intensity of 12,000 lux. After 60 days of growth in the greenhouse, plant height and stem diameter were measured, and the 3–5 fully expanded leaves from the upper part of the plants were collected, immediately frozen in liquid nitrogen, and stored at −80 °C for subsequent RNA extraction. Three biological replicates (independent plants) for WT or each transgenic line were analysed.

### 4.10. Drought Stress Treatment and Measurement of Traits

Based on preliminary experiments conducted in our laboratory, WT plants began to exhibit mild wilting after 10 days of water withholding and showed pronounced wilting after 15 days. Therefore, drought stress was imposed by withholding water for 15 days in this study. Two-month-old wild-type (WT) and transgenic plants were used for the drought treatment. Before treatment, soil water content was maintained at 65–70%, and drought stress was subsequently imposed by continuous withholding of water supply. After 10 days, leaves were collected from three individual WT plants for qRT-PCR analysis. To avoid disturbing the subsequent drought treatment, soil water content was not measured at the sampling point after 10 days of water withholding. After 15 days, plant phenotypes were observed and recorded, and 3–5 fully expanded leaves from the upper part of the plants were collected for determination of relative water content (RWC) and malondialdehyde (MDA) content. The leaf RWC [70] and MDA [71] content were measured according to previously reported methods, respectively.

### 4.11. Vector Construction and Poplar Transformation

The full-length cDNA of *PagPIP2;6A* was amplified from the cDNA of 84K poplar using the specific primers *PagPIP2;6A*-F and *PagPIP2;6A*-R and cloned into the pCXSN_flag vector containing the 35S promoter. The recombinant *35S*:*PagPIP2;6A* construct was stably transformed into 84K poplar via the *Agrobacterium*-mediated leaf disc method as previously described [72]. Under sterile conditions, leaves were harvested from 4-week-old sterile 84K poplar seedlings and cut into rectangular segments of 4–6 mm. Leaf explants were immersed in *Agrobacterium* suspension with an OD_600_ of 0.4 for 10 min. After incubation, leaf segments were taken out and blotted dry with sterile filter paper, then placed onto co-cultivation medium (1.991 g/L WPM + 0.5 g/L MES + 1 mg/L2,4-D + 100 μM AS). After incubation at 25 °C in the dark for 2 days, explants were transferred to selective medium and cultured under a 16 h light/8 h dark photoperiod at 25 °C. The selective medium was refreshed every 10 days. Once calli had formed, the explants were transferred to shoot induction medium for further culture. When the shoots reached approximately 3 cm, they were excised and transferred to rooting medium (2.41 g/L WPM + 30 g/L sucrose + 7.3 g/L agar + 400 mg/L cephalosporin + 12.5 mg/L kanamycin). Genomic PCR was carried out using primers 35S-F and *PagPIP2;6A*-R to amplify the CaMV35S-*PagPIP2;6A* fusion fragment, enabling preliminary screening of resistant seedlings to identify positive transgenic lines. Furthermore, qRT-PCR with primers *qPagPIP2;6A*-F and *qPagPIP2;6A*-R was applied to assess the transcript level of *PagPIP2;6A* in transgenic plants. All primer sequences are provided in Table S6.

### 4.12. Validation by Real-Time Quantitative PCR (RT-qPCR)

Total RNA was extracted from leaf samples using the Biospin Plant Total RNA Extraction Kit (Bioflux, Hangzhou, China) and reverse-transcribed into cDNA using the PrimeScript™ RT Kit (TaKaRa, Dalian, China). Gene expression was analyzed using a probe-free, SYBR Green dye-based Real-time quantitative polymerase chain reaction with SYBR Premix ExTaq™ (TaKaRa) on a qTOWER3G IVD Real-Time PCR System (Analytik Jena AG, Berlin, Germany). Each 10 μL reaction contained 5.0 μL of 2× SYBR Premix Ex Taq, 0.25 μL each of forward and reverse primers (10 μM), 1.0 μL of cDNA template, and 3.5 μL of RNase-free ddH_2_O. The amplification conditions were as follows: initial denaturation at 95 °C for 2 min, 35 cycles of 95 °C for 15 s and 55–60 °C for 30 s. The *Actin* gene of 84K poplar was used as an internal reference gene. Gene specific primers were designed using Primer Premier 5.0 software, and their specificity was verified using the Primer Check module in TBtools-II software (v2.485). Primer amplification efficiencies were determined using standard curves, and all primer pairs showed efficiencies within the acceptable range. All primers used are listed in Table S6. The relative expression levels between stressed and control samples were calculated using the 2^−ΔΔCt^ method [73]. Three biological replicates and three technical replicates were performed for each gene.

### 4.13. Statistical Analysis

All statistical analyses and graphing were performed using GraphPad Prism 9.0. Three biological replicates (independent plants) were included for each treatment and genotype. For RT-qPCR and the measurements of leaf RWC and MDA content, three technical replicates were performed for each biological replicate. Differences between two groups were analyzed using a two-tailed, unpaired Student’s *t*-test. Data are presented as mean ± standard deviation SD (*n* = 3), error bars indicate SD. Asterisks indicate significant differences between groups: * *p* < 0.05, ** *p* < 0.01, and *** *p* < 0.001.

## 5. Conclusions

In this study, 94 *PagAQP* genes were identified in the 84K poplar genome, of which the A and G subgenomes contained 46 and 48 members, respectively, and were divided into five subfamilies. Their chromosomal distribution, collinearity, gene structures, conserved motifs, substrate-recognition residues, promoter elements, and expression profiles revealed both conservation and divergence within the family. Most A/G allelic pairs were retained at corresponding genomic positions, and the analyzed allelic and paralogous pairs were predominantly subjected to purifying selection. Several *PagPIP* and *PagTIP* members were highly expressed in vascular tissues and exhibited changes in transcript abundance under nitrogen and drought treatments. The *35S*:*PagPIP2;6A* overexpression lines were shorter than wild-type plants but showed higher leaf relative water content and lower malondialdehyde content under drought stress. Furthermore, owing to the lack of additional drought-related physiological measurements, these results provide only preliminary evidence that *PagPIP2;6A* may be involved in responses to water deficit. Overall, this study provides a subgenome-level framework for understanding the evolution and expression of *PagAQPs* in hybrid poplar and identifies candidate genes for further functional validation.

## Figures and Tables

**Table 1:** Functional classification and distribution of predicted cis-acting elements of *PagAQP* across subfamilies.

Functional Category	Cis-Element	PagPIP	PagTIP	PagNIP	PagSIP	PagXIP	Total
Light response	light response	443	402	192	70	138	1245
Hormone response	abscisic acid response	90	79	19	14	29	231
	MeJA response	76	72	50	12	28	238
	gibberellin response	29	17	13	6	8	73
	auxin response	24	15	12	1	5	57
	salicylic acid response	15	11	15	1	2	44
	zein metabolism regulation	10	6	3	2	5	26
Abiotic stress	drought response	10	13	5	4	3	35
	low-temperature response	17	9	9	5	2	42
	anaerobic induction	72	60	57	13	25	227
Biotic stress	wound-responsive element	1	1	0	0	0	2
	defense and stress response	5	12	18	2	6	43
Growth and development	tissue elements	32	22	23	4	11	92
	cell cycle	4	2	2	0	0	8
Transcription regulation	MYB binding site	18	15	3	2	7	45
	cis-acting regulatory element	11	17	6	0	1	35
Circadian rhythm	circadian control	8	5	2	0	5	20
Other elements	ATBP-1	8	8	6	3	2	27
	protein binding site	0	1	3	1	3	8
	Total	873	767	438	140	280	2498

## Data Availability

All data can be found in the article or in the Supplementary Materials.

## Supplementary Materials

The following supporting information can be downloaded at: https://www.mdpi.com/article/10.3390/plants15203167/s1, Table S1: Characteristics of the 94 *PagAQPs* identified from the 84K poplar genome; Table S2: The Ka/Ks ratios of *PagAQP* allele pairs in 84K poplar; Table S3: The Ka/Ks ratios of intra-subgenomic paralogous gene pairs of *PagAQP* in 84K poplar; Table S4: Amino acid compositions of the NPA motifs, Ar/R selectivity filters, and Froger’s positions of PagAQPs; Table S5: Analysis of cis-acting element of *PagAQPs*; Table S6: Primers used in this study.
